# Outcomes and management of primary tumors in metastatic MSI/dMMR colorectal cancer patients treated with immune checkpoint inhibitors: a cohort study

**DOI:** 10.1016/j.esmogo.2026.100319

**Published:** 2026-03-14

**Authors:** L. Mas, C. Grosnon, T. Samaille, A. Dardenne, Y. Parc, J. Lefèvre, R. Cohen, T. André

**Affiliations:** 1 Department of Medical Oncology, Sorbonne University, Hôpital Saint-Antoine, AP-HP, Paris, France; 2 Department of Digestive Surgery, Sorbonne University, Hôpital Saint-Antoine, AP-HP, Paris, France; 3 INSERM UMRS 938, Équipe Instabilité des Microsatellites et Cancer, SIRIC CURAMUS, Saint-Antoine Research Center, Paris, France

**Keywords:** primary tumor resection, metastatic colorectal cancer, immune checkpoint inhibitors, MSI-H

## Abstract

**Background:**

The optimal management of primary tumor (PT) in microsatellite instability-high metastatic colorectal cancer patients treated with immune checkpoint inhibitors (ICIs) remains unclear.

**Patients and methods:**

We retrospectively analyzed a prospective, single-center cohort to assess PT outcomes in this setting.

**Results:**

Among 210 patients, 21% received first-line ICI; we focused on 33 patients (16%) without prior PT resection at ICI initiation. Early progressive disease (<6 months) occurred in 10 patients (5%), with eight deaths (<1 year); two underwent surgery for symptomatic PT. Among the remaining 23 patients (11%) with disease control (≥6 months), 15 had PT resection due to clinical events (*n* = 5) or for non-symptomatic reasons (*n* = 10), including seven with pathological complete response and eight with residual tumor. Eight patients had no PT resection, including two with metastatic and/or local progressive diseases. After a median 75-month follow-up, only six patients (3%) remained progression-free with PT in place and no residual endoscopic tumor.

**Conclusions:**

If PT is left in place at ICI initiation, close monitoring is required due to its specific evolution. Resection may be indicated for symptomatic stenosis. Prospective trials are needed to define the role of endoscopic monitoring and indication of PT resection in first-line ICI.

## Highlights

• Some 16% of dMMR/MSI-H mCRC patients had unresected primary tumor (PT) at ICI initiation.
• Some 40% of patients with unresected PT achieved pathological complete response.
• Median time to PT resection after ICI was 15 months.
• PT surgery may be needed for obstruction, perforation, or symptomatic stenosis.
• Close PT monitoring is recommended; prospective trials are needed to guide resection.

## Introduction

Colorectal cancer (CRC) ranks as the third most commonly diagnosed cancer and the second leading cause of cancer-related mortality worldwide.^1^ Among its molecular subtypes, microsatellite instability-high (MSI-H) or mismatch repair-deficient (dMMR) CRC, characterized by defects in DNA repair, accounts for ∼5%-7% of metastatic CRC (mCRC) cases.^2^ This subtype has recently emerged as a distinct therapeutic entity, as immune checkpoint inhibitors (ICIs) have demonstrated superiority over chemotherapy as first-line treatment of metastatic disease, based on the results of the KEYNOTE-177 and CheckMate 8HW phase III trials.^3^^,^^4^ In patients with microsatellite-stable (MSS) or mismatch repair-proficient (pMMR) mCRC, randomized phase III trials such as iPAC and CAIRO4 have shown that primary tumor (PT) resection followed by chemotherapy and targeted therapy offers no survival benefit compared with systemic treatment alone in the setting of an asymptomatic PT with synchronous unresectable metastases.^5^^,^^6^ Consequently, PT resection in pMMR mCRC should be reserved for patients with symptomatic PT. With the exception of symptomatic PT requiring surgery, including bowel obstruction associated with ICI response, a rare treatment-related complication, the outcomes and optimal management strategy of the PT during ICI therapy in mCRC remains to be defined.

## Patients and methods

We conducted a retrospective analysis of a prospective single-center cohort study of patients treated at Saint-Antoine Hospital in Paris, France. Data were obtained from the ImmunoMSI database and patient medical records. Non-objection to the use of routine care data and database information was obtained from all study patients, except those who were deceased. This cohort was approved by the ethics committee (No. 2020–CER 2020-6). Our study cohort included patients >18 years of age, who had received at least one injection of ICIs [anti-programmed cell death protein 1 (anti-PD-1) or anti-programmed death-ligand 1 (anti-PD-L1) ± anti-cytotoxic T-lymphocyte-associated protein-4 (anti-CTLA-4)] for a dMMR/MSI-H mCRC. The dMMR/MSI-H status was determined locally through immunohistochemistry and multiplex polymerase chain reaction. Categorical variables were described by their number and frequency. Progression-free survival (PFS) was defined as the time from ICI initiation to disease progression or death. Radiologic response was assessed by expert radiologists using Response Evaluation Criteria in Solid Tumors (RECIST) 1.1 and immune RECIST (iRECIST). Survival analyses were carried out using the Kaplan–Meier method.

We retrospectively identified all cases of mCRC with an unresected PT at the time of ICI initiation within the prospective ImmunoMSI cohort, which included all patients with dMMR/MSI mCRC treated with ICIs at Saint-Antoine Hospital between May 2015 and June 2024. Clinical and pathological data were collected, including patient demographics, tumor characteristics (PT location, metastatic sites, and presence of a digestive stoma), prior treatments, and outcomes under ICI therapy and subsequent lines of treatment. For patients with disease control lasting ≥6 months, in whom assessment of PT natural history under ICI therapy was relevant, particular attention was given to the clinical course and management of the PT. Clinical indications for PT resection were assessed, and histopathological reports from resected specimens and/or endoscopic biopsies, as well as surveillance colonoscopy reports, were reviewed to evaluate local tumor evolution, including residual tumor, stenosis, or other local complications.

## Results

The prospective ImmunoMSI cohort included 210 patients with dMMR/MSI mCRC treated with ICI, with a median follow-up of 75 months. Thirty-three patients (16%) had a PT in place at ICI initiation (Table 1, Supplementary Figure S1, available at https://doi.org/10.1016/j.esmogo.2026.100319). No significant differences were observed in patients or tumor characteristics; however, the absence of prior PT resection was significantly associated with receiving ICI as first-line treatment (36% versus 18%, *P* = 0.03). Among these 33 patients without prior PT resection, 10 experienced early progressive disease (PD ≤6 months), while 23 achieved disease control ≥6 months (iRECIST evaluation). A total of 15 of the 23 patients subsequently underwent secondary PT resection, including 9 segmental colectomies, 2 rectal resections, and 4 sub-total colectomies. Patient characteristics stratified by disease control and PT resection after receiving ICI are presented in Table 2. Notably, patients achieving disease control and undergoing PT resection had a significantly higher rate of dual ICI therapy (73%). Of the 10 patients with early PD, two underwent surgery for symptomatic occlusive syndrome due to PT progression, and eight died shortly afterwards without PT-related events.

**Table 1:** Patient and disease characteristics according to PT status at ICI initiation

	Overall^a^	Prior PT resection *n* = 177^a^	No prior PT resection *n* = 33^a^	*P* value^b^
Age at diagnosis (median), years	61 (50-71)	61 (51-71)	58 (49-66)	0.5
Sex, *n* (%)				0.4
Male	94 (45)	77 (44)	17 (52)	
Female	116 (55)	100 (56)	16 (48)	
Stage at initial diagnosis, *n* (%)				<0.001
I-III	110 (52)	108 (61)	2 (6)	
IV	100 (48)	69 (39)	31 (94)	
Tumor location, *n* (%)				0.091
Right colon	132 (63)	113 (64)	19 (58)	
Transverse colon	5 (2)	3 (2)	2 (6)	
Left colon	53 (25)	47 (27)	6 (18)	
Rectum	20 (9)	14 (8)	6 (18)	
Prior line(s) of treatment, *n* (%)				0.030
0	44 (21)	32 (18)	12 (36)	
1	58 (28)	48 (27)	10 (30)	
>1	108 (51)	97 (55)	11 (33)	
Immunotherapy type, *n* (%)				0.8
Anti-PD-1 monotherapy	114 (54)	97 (55)	17 (52)	
Anti-PD-1 + anti-CTLA-4	95 (45)	79 (45)	16 (48)	
Immunotherapy + chemotherapy	1 (0.5)	1 (1)	0 (0)	
Performance status, *n* (%)				0.064
0	88 (42)	79 (45)	9 (27)	
1-2	122 (58)	98 (55)	24 (73)	
Number of metastatic sites, *n* (%)				0.9
1	93 (44)	78 (44)	15 (45)	
>1	117 (56)	99 (56)	18 (55)	
BRAF status, *n* (%)				0.8
Mutated	48 (23)	41 (23)	7 (21)	
Wild-type	148 (70)	125 (71)	23 (70)	
NA	14 (7)	11 (6)	3 (9)	
KRAS/NRAS status, *n* (%)				0.2
Mutated	76 (36)	62 (35)	14 (42)	
Wild-type	116 (55)	102 (58)	14 (42)	
NA	18 (9)	13 (7)	5 (15)	

CTLA-4, cytotoxic T-lymphocyte-associated protein-4; ICI, immune checkpoint inhibitor; NA, not available; PD-1, programmed cell death protein 1; PT, primary tumor.

a Median (Q1, Q3); *n* (%).

b Wilcoxon rank sum test; Pearson’s chi-square test; Fisher’s exact test.

**Table 2:** Patient characteristics stratified by disease control and PT resection

	Early PD *n* = 10^a^	Disease control without PT resection *n* = 8^a^	Disease control with PT resection *n* = 15^a^	*P* value^b^
Age (median), years	57 (44-72)	62 (56-65)	53 (48-65)	0.6
Unknown	0	1	1	
Sex, *n* (%)				0.5
Male	6 (60)	5 (63)	6 (40)	
Female	4 (40)	3 (38)	9 (60)	
Tumor side, *n* (%)				0.6
Right colon	6 (60)	6 (75)	7 (47)	
Transverse colon	0 (0)	0 (0)	2 (13)	
Left colon	2 (20)	0 (0)	4 (27)	
Rectum	2 (20)	2 (25)	2 (13)	
Prior line(s) of treatment, *n* (%)				0.8
0	3 (30)	3 (38)	6 (40)	
1	4 (40)	3 (38)	3 (20)	
>1	3 (30)	2 (25)	6 (40)	
Prior digestive stoma	2 (20)	1 (13)	4 (27)	0.9
Immunotherapy type, *n* (%)				0.032
Anti-PD-1 monotherapy	7 (70)	6 (75)	4 (27)	
Anti-PD-1 + anti-CTLA-4	3 (30)	2 (25)	11 (73)	
Immunotherapy + chemotherapy	0 (0)	0 (0)	0 (0)	
Performance status, *n* (%)				0.2
0	1 (10)	4 (50)	4 (27)	
1-2	9 (90)	4 (50)	11 (73)	
Number of metastatic sites, *n* (%)				0.5
1	6 (60)	4 (50)	5 (33)	
>1	4 (40)	4 (50)	10 (67)	
BRAF status, *n* (%)				0.7
Mutated	3 (30)	2 (25)	2 (13)	
Wild-type	7 (70)	5 (63)	11 (73)	
NA	0 (0)	1 (13)	2 (13)	
KRAS/NRAS status, *n* (%)				0.3
Mutated	5 (50)	5 (63)	4 (27)	
Wild-type	5 (50)	2 (25)	7 (47)	
NA	0 (0)	1 (13)	4 (27)	

CTLA-4, cytotoxic T-lymphocyte-associated protein-4; NA, not available; PD, progressive disease; PD-1, programmed cell death protein 1; PT, primary tumor.

a Median (Q1, Q3); *n* (%)

b Kruskal–Walli’s rank sum test; Fisher’s exact test.

**Figure 1:**
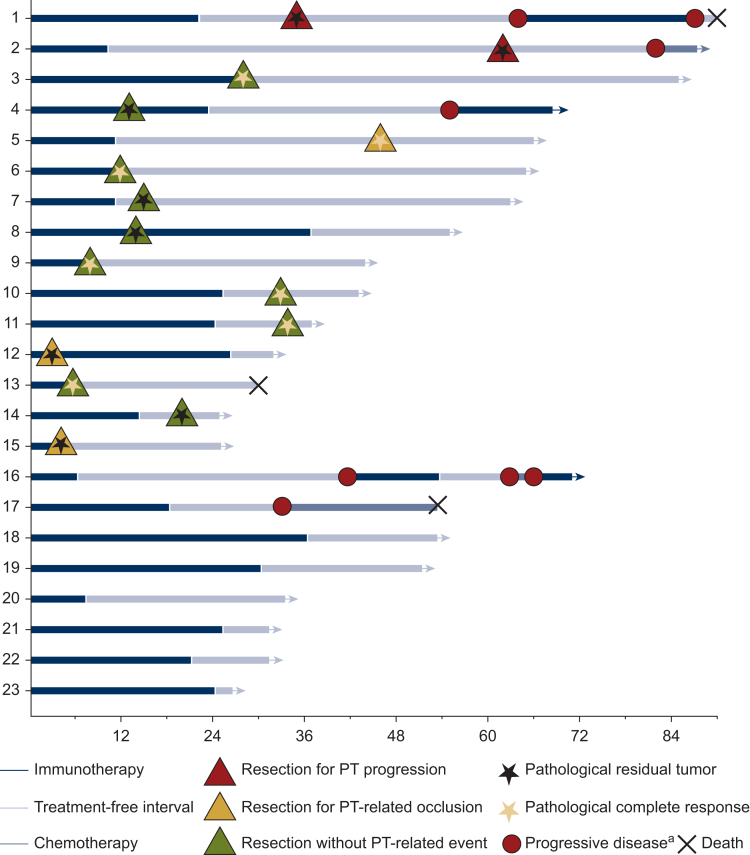
**Clinical course of 23 patients with PT in place at ICI initiation and disease control ≥6 months.** ICI, immune checkpoint inhibitor; PT, primary tumor. ^a^Progressive disease: metastatic progression or isolated unresectable PT progression.

**Figure 2:**
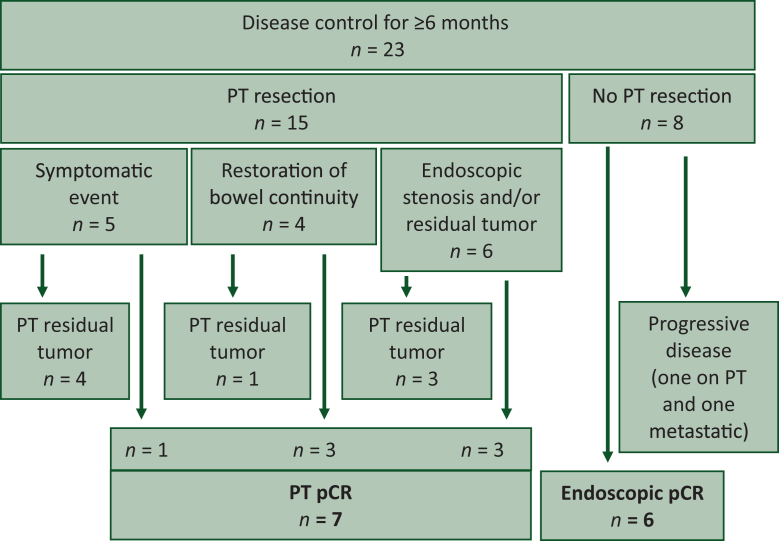
**Outcomes in patients without prior PT resection and disease control for ≥6 months.** pCR, pathological complete response; PT, primary tumor.

The clinical course of the 23 patients with disease control ≥6 months is illustrated in Figure 1. Among these, 15 patients underwent PT resection with a median of 15 months from ICI initiation (range 3-62 months; Figure 2). Five patients underwent surgery due to PT-related events: two developed upstream occlusion without PD at 3 and 4 months after ICI initiation (patients 12 and 15 with tumoral stenosis on PT), two had asymptomatic isolated local PT progression 13 and 51 months after ICI discontinuation (patients 1 and 2), and one experienced post-endoscopic colonic perforation above a non-tumoral fibrous stenosis 34 months after ICI discontinuation (patient 5). The remaining 10 patients underwent surgery while asymptomatic 5-34 months after ICI initiation, based on investigator decision. Indications included endoscopic stenosis (*n* = 5), restoration of bowel continuity (*n* = 4), and residual tumor confirmed by endoscopic biopsy (patient 4). Pathological analysis revealed complete pathological response (pCR) on PT specimen in seven patients, while eight had residual tumor on surgical specimen, including both patients operated for isolated PT progression (patients 1 and 2) and the two patients with tumor-related occlusion (patients 12 and 15).

At the time of PT surgery, eight patients were still receiving ICI; only one experienced PD 42 months after PT resection and 32 months after ICI discontinuation (patient 4). Among the seven patients undergoing PT resection after ICI discontinuation, none resumed treatment postoperatively, and only the two patients operated on for isolated late PT progression experienced secondary PD 20 and 29 months after surgery (patients 1 and 2). Of the five patients with endoscopic stenosis and biopsy-proven pCR, four had pCR confirmed on pathologic analysis, and one had residual tumor.

No difference was seen in ICI duration or radiological partial response rate when comparing patients with versus without PT resection, or those receiving dual versus monotherapy (Supplementary Tables S1 and S2, available at https://doi.org/10.1016/j.esmogo.2026.100319). Four patients achieved radiological complete response; all had undergone PT resection, either for endoscopic stenosis (*n* = 2) or for residual endoscopic tumor and restoration of bowel continuity.

Among the eight patients who did not undergo PT resection, six had negative biopsies at last endoscopic follow-up and remained free of PD 26-53 months after ICI initiation and 2-26 months after ICI discontinuation. The remaining two patients experienced PD: one had residual tumor on the last endoscopic biopsy and developed unresectable isolated PT progression 33 months after ICI discontinuation, while the other, lacking endoscopic surveillance, presented with metastatic-only PD 15 months after ICI discontinuation. Overall, of the 23 patients with PT in place with disease control ≥6 months, 13 achieved pCR of the PT, either on surgical specimen or endoscopic biopsy, and none of these 13 experienced PD.

## Discussion

After a median follow-up of 75 months in this cohort of 210 patients with dMMR/MSI-H mCRC treated with ICIs, 33 (16%) had no prior PT resection at ICI initiation. Among these, 13/33 (40%) achieved pCR on the PT (either after surgical resection or on endoscopic evaluation), and only six patients (3% of the total cohort) had their PT in place and in pCR at the data cut-off.

This highlights the contrast between the high effectiveness of ICIs in this population and the small number of patients who still have their PT in place at the database lock. To date, high pCR rates observed with neoadjuvant ICI have prompted investigations into adaptative watch-and-wait strategies for patients with MSI-H/dMMR non-metastatic CRC.^7^, ^8^, ^9^, ^10^ By analogy, a watch-and-wait approach for the colorectal PT may also be considered in metastatic disease when the tumor is in place and largely asymptomatic at the time of ICI initiation.

In this cohort, having PT in place at ICI initiation was significantly associated with the first-line setting, which accounted for only 21% of patients, partially explaining the low number of patients with unresected PT at the start of ICI treatment. It remains unclear whether ICIs are equally effective on the PT in a metastatic versus a non-metastatic setting. Bowel obstruction associated with ICI response has been previously reported as an important complication. Notably, obstruction secondary to ICI-induced PT response with stenosis, rather than PD, is now well recognized.^11^^,^^12^

Among the 33 patients with PT in place at ICI initiation, we observed five cases requiring surgery for occlusive syndrome or perforation: two inflammatory tumors without PD, two with local tumoral PD, and one perforation over a fibrotic stenosis without residual tumor. Additionally, five patients underwent PT resection for asymptomatic endoscopic or radiologic stenosis, three of whom had preoperative negative biopsies and were found to have pCR on the surgical specimen; the remaining two had residual tumor. These findings highlight, as previously reported, that stenosis after ICI treatment without tumoral cells on the PT is not rare. Pellat et al.^13^ showed that patients with Lynch syndrome treated with ICI for dMMR/MSI-H mCRC are at risk of developing metachronous dMMR/MSI-H cancers and pre-neoplastic colorectal polyps. In that study, 8% of patients developed metachronous cancers, and 39% of patients undergoing colonoscopy during follow-up had pre-neoplastic colorectal polyps.

All these data underline the importance of dedicated long-term colonoscopic follow-up in patients with dMMR/MSI-H mCRC, even after successful ICI treatment. A limitation of our series is that most patients had received extensive prior treatment with chemotherapy ± targeted therapy before ICI treatment, with only 21% receiving first-line treatment. Current standard treatment of these patients is ICI, based on the KEYNOTE-177 and CheckMate 8HW phase III data.^3^^,^^14^ The low number of patients without prior PT resection represents another limitation of this study, with only 16% of the total cohort starting ICI with their PT in place. This rate, however, is consistent with the known biology of dMMR/MSI-H CRC, as this phenotype is approximately twice as common in localized compared with metastatic disease,^2^ which explains that more than half of the patients in our cohort had initial stage I-III tumors. This distribution is similar to that reported in the aforementioned phase III trials, where 50% and 43% of patients with stage IV disease at diagnosis; however, these trials did not report the status of the PT at inclusion (resected or unresected) or its outcomes during follow-up. Among patients with synchronous stage IV mCRC in our cohort, 69% had their PT resected before ICI initiation. This proportion appears consistent with the existing date, considering that 15%-30% of patients with synchronous mCRC undergo curative intent surgery,^15^^,^^16^ and that a significant proportion of our patients were treated during a period when PT resection in the setting of unresectable metastatic disease was widely carried out, reported in up to 60% of cases in large series from the 2010s,^17^ before the results of dedicated phase III trials^5^^,^^6^ were published.

Given these considerations, future prospective studies are needed to evaluate the outcome of unresected PT in patients receiving first-line ICI therapy for dMMR/MSI-H mCRC. This would help to better define the optimal frequency of colonoscopic monitoring and determine the appropriate management decisions for asymptomatic stenosis and residual lesions detected on endoscopy.
