# Supplementary figures and images for: Outcomes and management of primary tumors in metastatic MSI/dMMR colorectal cancer patients treated with immune checkpoint inhibitors: a cohort study
Source: ESMO Gastrointest Oncol. 2026 Mar 14;12:100319. doi: 10.1016/j.esmogo.2026.100319 (PMC13000474; doi:10.1016/j.esmogo.2026.100319)

**Supplementary Figure S1**. Study flowchart showing patient selection.


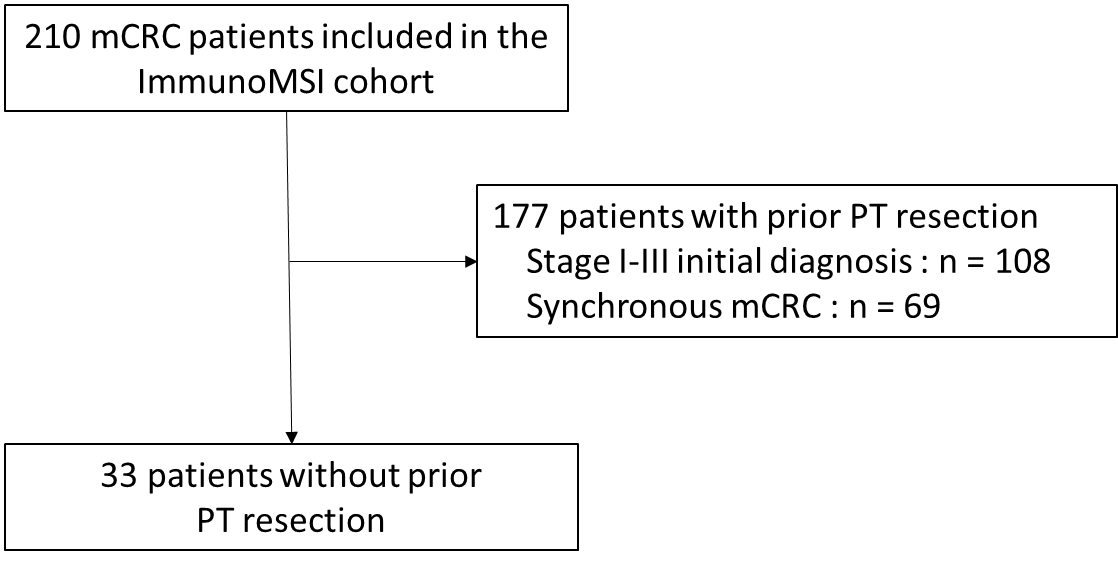

Supplement: Supplementary Figure 1 [file mmc1.docx]
