# Supplementary material for: Outcomes and management of primary tumors in metastatic MSI/dMMR colorectal cancer patients treated with immune checkpoint inhibitors: a cohort study
Source: ESMO Gastrointest Oncol. 2026 Mar 14;12:100319. doi: 10.1016/j.esmogo.2026.100319 (PMC13000474; doi:10.1016/j.esmogo.2026.100319)
Supplement: Supplementary Table 1 [file mmc2.docx]

**Supplementary Table S1.** Outcomes under ICI therapy according to secondary PT resection status in 23 patients with disease control ≥6 months.

|  | PT resection *n* = 15 | No PT resection *n* = 8 | *P*-value^1^ |
| --- | --- | --- | --- |
| ICI duration, *months, median (Q1-Q3)* | 14 (10-25) | 23 (13-27) | 0.5 |
| Radiological  partial response, *n* (%) | 13 (87) | 7 (88) | >0.9 |
| Radiological  complete response, *n* (%) | 4 (27) | 0 (0) | 0.3 |

*^1^* Wilcoxon rank sum test; Fisher’s exact test
