# Supplementary material for: Outcomes and management of primary tumors in metastatic MSI/dMMR colorectal cancer patients treated with immune checkpoint inhibitors: a cohort study
Source: ESMO Gastrointest Oncol. 2026 Mar 14;12:100319. doi: 10.1016/j.esmogo.2026.100319 (PMC13000474; doi:10.1016/j.esmogo.2026.100319)
Supplement: Supplementary Table 2 [file mmc3.docx]

**Supplementary Table S2.** Outcomes under ICI therapy according to immunotherapy type in 23 patients with disease control ≥6 months.

|  | Anti-PD(L)1 monotherapy *n* = 10 | Anti-PD1 + Anti-CTLA4 *n* = 13 | *P*-value^1^ |
| --- | --- | --- | --- |
| ICI duration, *months, median (Q1-Q3)* | 21 (8-30) | 21 (11-25) | 0.6 |
| Radiological  partial response, *n* (%) | 9 (90) | 11 (85) | >0.9 |
| Radiological  complete response, *n* (%) | 0 (0) | 4 (31) | 0.10 |
|  | | | |
|  | | | |

*^1^* Wilcoxon rank sum test; Fisher’s exact test
